# Exploring inequities in skilled care at birth among migrant population in a metropolitan city Addis Ababa, Ethiopia; a qualitative study

**DOI:** 10.1186/s12939-014-0110-6

**Published:** 2014-11-25

**Authors:** Alemnesh H Mirkuzie

**Affiliations:** Center for International Health, University of Bergen, Årstadv 21, Overlegedanielsenshus, 5020 Bergen

**Keywords:** Addis Ababa, Access, Antenatal, Disrespectful care, Equity, Ethiopia, Home birth, Missed opportunities, Postpartum care, Quality care, Skilled birth care, Utilization

## Abstract

**Introduction:**

Ethiopia records high levels of inequity in skilled birth care (SBC), where the gaps are much wider among urban migrant women. An intervention project has been conducted in Addis Ababa, intending to improve quality and to ensure equitable access to maternal and newborn care services. As part of the project, this study explored the inequities in maternal health care among migrant women in Addis Ababa, Ethiopia.

**Methods:**

A qualitative community based study was conducted from April to May 2014 among 45 purposefully selected internal migrant women. Eleven women who give birth at home and eight who gave birth at health facility in the last year preceding the study participated in in-depth interviews. Four primiparas’ young women, 18 women who have children and four grandmothers participated in focus group discussions. Guides were used for data collection. Using framework and content analysis three themes and four sub-themes emerged.

**Results:**

According to the informants, patterns of service utilization varied widely. Antenatal care and infant immunization were fairly equally accessed across the different age groups of informants in their most recent birth irrespective of where they gave birth, yet obvious access gaps were reported in SBC and postpartum care. There were missed opportunities to postpartum care. Only few women had received postpartum care despite, some of the women delivering in the health facility and many visiting the health facilities for infant immunization. The four emerged sub-themes reportedly influencing access and utilization of SBC were social influences, physical access to health facility, risk perceptions and perceived quality of care and disrespect. Of these social, structural and health system factors, informants presented experiences of disrespectful care as a powerful deterrent to SBC.

**Conclusions:**

Migrant women constitute disadvantaged communities in Addis Ababa and have unequal access to SBC and postpartum care. This happens in the backdrop of fairly equitable access to antenatal care, infant immunization, universal health coverage and free access to maternal and newborn care. Addressing the underlying determinants for the inequities and bridging the quality gaps in maternal and newborn services with due emphasis on respectful care for migrant women need tailored intervention and prioritization.

**Electronic supplementary material:**

The online version of this article (doi:10.1186/s12939-014-0110-6) contains supplementary material, which is available to authorized users.

## Introduction

Skilled birth care (SBC) and Emergency Obstetric and Neonatal Care are high impact, cost effective priority interventions recording the highest return in improving maternal and neonatal outcomes in resource poor settings [1-5]. For these interventions to succeed there should be an improved coverage, equitable access and the services should have good quality.

There are several factors determining access to maternity care services in general and access to SBC in particular. The three delays model is extensively used to explain both demand and supply side constraints responsible for poor access to SBC [6]. According to this model, the ‘first delay’ is a demand side delay that happens when there is a failure in the part of the women to recognize the need for SBC. The ‘second delay’ occurs when there is a delay to reach health care facility due to structural, social and economic barriers. The ‘third delays’ is when women fail to receive prompt care in the health facility and is concerned with quality of care.

Access to SBC can also be determined by how the women perceive the quality of care they receive during pregnancy, labour and delivery [7-11]. Most recent review that compiled data from poor resource settings documented the widespread practice of disrespectful care and abusive treatment by health care workers when women seek SBC and the power of such experiences in undermining access to SBC in their subsequent deliveries [11]. The WHO recently has made a call for action to end disrespectful SBC from policy to practice [12]. The call highlights that among others, women of low socioeconomic status, migrant women and women of ethnic minorities are common victims of disrespectful SBC.

Ethiopia is one of the countries having low proportions of women receiving SBC, although progresses have been made in recent years. According to the 2011 Demographic and Health Survey (DHS) report, only 10% of the births in Ethiopia are taking place at the health facilities [13]. Not only that, Ethiopia is reported to be the most inequitable country with respect to access to SBC with huge disparities across and within the regions. In Addis Ababa 84% of the women receive SBC, whereas only 6% of the women in the Southern region receive SBC [13]. Urban women are in general more privileged in terms of access to SBC than their rural counterparts [14,15]. Even within the urban strata women with high household economy are over six times more likely to access birth care at health facility than women with low household economy in Addis Ababa [15]. This inequity happens despite free provision of maternal and newborn care services at primary health care units and universal health services coverage [16]. According to the Ethiopian Health Care Financing Reforms, maternal and newborn care services are fee exempted services at all public primary care facilities [17,18]. In public hospitals, although these are payable services there is a fee waiver system for poor women who should access hospital care.

Ethiopia has recorded fast economic development that significantly increased rural to urban migration of people in search for better economic opportunities, where these are marked in Addis Ababa, the capital. Yet, economic transitions and fast urbanization do not happen without a cost. Countries recording fast economic growth and development often have challenges to bridge disparities in access to health care among the different population segments. Taking the case of China, a country where fast economic development are happening for years; rural–urban migrants (floating population) have unequal access to health care compared to resident population. Such disparities are more prevalent among the less educated, women and children than other groups. Migrant women have lower level of perinatal care services utilization and higher risks of adverse obstetric and neonatal outcomes than resident women. Structural, social and behavioural factors are implicated for the inequitable access [19]. Two studies in Ethiopia have reported that there is an association between rural–urban migration and poor health outcomes and increased risks/vulnerabilities to HIV [20,21]. Yet, evidence is lacking on access to SBC among migrant women in Ethiopia in general and in Addis Ababa in particular.

Despite the wide inequities in access to SBC in Addis Ababa, little has been done to identify the underlying determinants and to intervene on the gaps. To contribute to the efforts in improving maternal and newborn health outcomes in Addis Ababa, a capacity building intervention project has been conducted in collaboration with the Centre for International Health, University of Bergen, the Addis Ababa City Council Health Bureau and the Addis Ababa University, Ethiopia has been conducted in Addis Ababa. The project intends to improve the quality of basic emergency obstetric and neonatal care in Addis Ababa and to ensure equitable access. The project has focused both on the supply and on the demand side. On the supply side, emphasis has been given for improving the quality of maternal and neonatal care in 10 randomly selected public primary health centers through intensive hands-on skills training using low cost and low-tech simulators (MamNatalie and NeoNatalie). Developing and implementing a management protocol for health center use was the other intervention. For bridging the equity gaps, the project targeted public health centers where the majority of poor and disadvantaged women are accessing emergency obstetric and neonatal care services free of charge. The demand side focus of the project was exploring access to maternal and newborn care services among women of disadvantaged background. Therefore, this study explored the inequities in access to SBC among internal urban–rural migrants in Addis Ababa the capital of Ethiopia’ using a qualitative approach. Most studies concerning inequity in access to SBC often focus on the magnitude, but not what lies behind the numbers.

## Methods

### Settings

Addis Ababa city having a population of about 3.5 million people is administratively divided into10 sub cities and Woredas. The Woreda’s are the smallest administrative units. Among the regions in Ethiopia, Addis Ababa has the highest proportion of women receiving SBC with great variation across the 10 sub-cities. Gulele is one of the sub-cities reporting low proportions of women receiving SBC (Unpublished Health Management Information System report from Addis Ababa City Administration Health Bureau). Although, the population of Addis Ababa is reported to be heterogeneous in terms of religion, ethnic and socioeconomic diversities; more homogeneity seen in some parts of the city especially those areas inhabited by migrants from remote rural areas. Migrant population from Gamo ethnic group are predominantly living in Gulele sub-city Woreda six with limited mixing with the other communities in the areas. Home birth is a common practice in this community. These marginalized groups of people are economically disadvantaged depending heavily on handcraft (weaving) and fetching and selling firewood for a living. Women in this community often have low education where all these factors could play a role in their decision where they should give birth.

Addis Hiwot is the catchment health center, where the neighbourhood communities access a range of services including maternal and newborn care free of charge. There were 12 providers offering maternal and newborn care services but only one had training on emergency obstetric and newborn care. Retained placenta is a common postpartum obstetric complication in the health center after home birth. There are urban health extension workers providing home-based services with much emphasis on health promotion. With respect to maternal health, the urban health extension workers are primarily working on demand creation for infant immunization, antenatal (ANC), SBC and postpartum (PPC).

### Study design

A community based qualitative study was conducted from April to June 2014 in Woreda six Gulele sub-city that has large urban–rural migrant population. The study employed individual in-depth interviews and focus group discussions (FGDs). Wutich and colleagues who compared how FGDs perform against individual responses on sensitive topics make a recommendation that combining different methods of data collection would increase credibility of the study, as each data collection method would give unique contexts [22]. In this study the individual in-depth interviews as the major data collection method was used to explore barriers and facilitator to access SBC and to capture individual experiences. Findings from preliminary analysis of the in-depth interviews had made it clear that having intergenerational views and exploring its influence on access to SBC would complement the study findings. In the FGD, experiences of SBC utilization across the different generations of internal migrant women and their views on access to SBC were explored. Studies show intergenerational influences on seeking SBC in particular grandmothers and mothers in law are often having high authorities in the family concerning childbirth owing to their lived experiences and wisdom [9,10]. According to Solomon and colleagues, elderly women in Ethiopia “As opinion leaders especially on matters related to labour, their position influences the care seeking behaviour of particularly young mothers” [10].

### Selection of study participants

**Figure 1:**
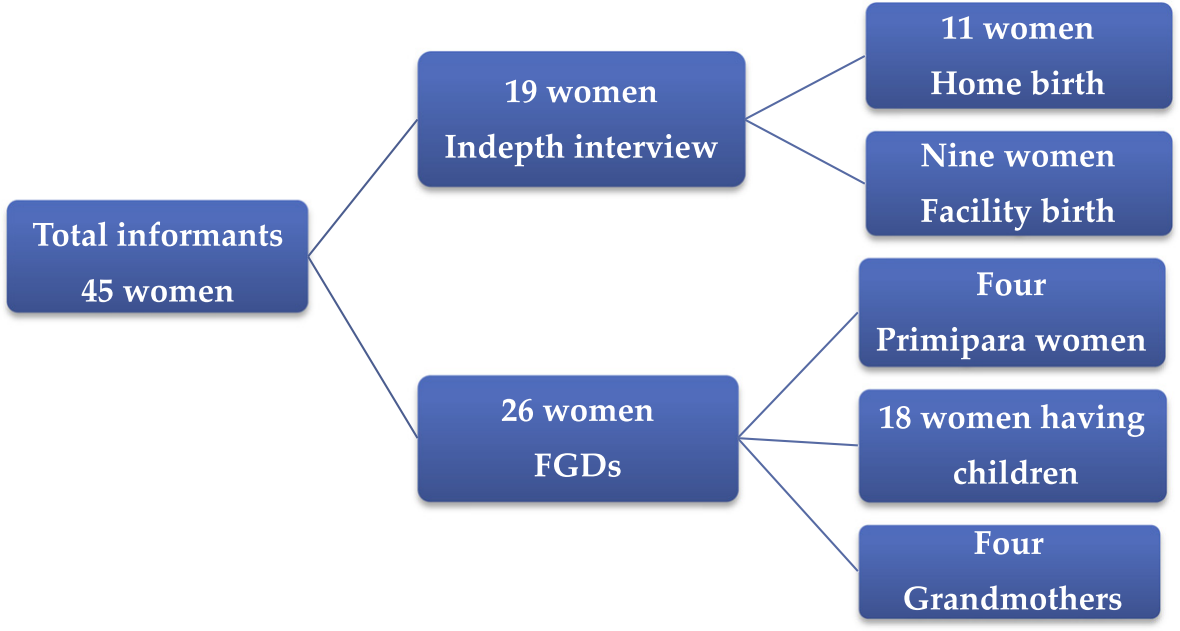
**Study flow chart.**

Nineteen migrant women who gave birth within the past year prior to the study were purposefully selected for in-depth interviews. The inclusion criteria were those who gave birth within the past year, who were available on the date the data was collected and those who gave consent to be interviewed. Women who speak language not known by the principal investigator and by the translator were excluded. Of 21 women who were approached for the in-depth interviews, two did not participate due to language barriers (Figure 1). Following the preliminary analysis of the in-depth interviews, 28 women with diverse procreative background were purposefully selected and invited to participate in the FGDs. Of which two did not show up and 26 women i.e. four primiparas’ young women, 18 women who have children and four grandmothers participated in four FGD sessions (Figure 1).

Except four all the 45 informants were from the Gamo tribe. Sixteen women had extended families and six were living with mothers in law while the rest were having some other relatives living together as family members. Twenty women were illiterate. For the women with some schooling, the maximum was 10th grade completed. The number of deliveries per woman ranged from one to eight while age of the last (index) child ranged from less than a week to 20 years.

### Data collection

The urban health extension workers familiar with the villages and the women were instrumental to identify and approach eligible women for the interviews and the FGDs. All the in-depth interviews took place in informants’ homes. Home is a natural setting for the women to feel free to respond to the interview questions and it would also offers an opportunity for the data collector to understand the context that the woman is living [23]. An interview guide was first developed in English then translated to Amharic, which is the national language. The guide includes questions to explore the rationale for giving birth at home/at health facility and to explore the underlying influencing factors (See attached Additional file 1).

Following the in-depth interviews, four FGDs were conducted. There were seven informants each in two FGDs sessions and eight informants each in two FGDs sessions. Informants were randomly distributed in the FGD sessions. A guide that was developed following the preliminary analyses of the in-depth interviews was used for the FGDs. In the discussions all informants were made to express their views while respecting each other’s viewpoint. In almost all the FGD sessions’ group consensus were reached, although different views on place of birth were surfaced.

The principal investigator who is a native speaker of Amharic language conducted all the interviews and the FGDs. Amharic is the national language spoken fluently by the majority of the people in Ethiopia. However, six informants were not well conversant with Amharic and hence local interpreter was used. Questionnaires in the front page of the interview and the FGD guides were used to collect data on demographic and obstetric characteristics of informants and on utilization of maternal and newborn care services.

Member checking was an integral part of the data collection procedure in order to increase credibility (internal validity) of the data [24]. During the in-depth interviews and the FDGs the principal investigator was constantly checking her understanding of the phenomenon by paraphrasing what the informants have said. Further member checking were done at the end of each individual interviews and FGDs, where informants were asked to verify the information summarized by the principal investigator was accurately reflecting their views and experiences about SBC.

The interviews and the FGDs lasted from 45 to 60 minutes. All the in-depth interviews and the FGDs were audio taped and notes were taken.

### Data analyses

The interviews and the FGDs were first transcribed verbatim, and then translated to English. Due to time constraints the principal investigator was not able to transcribe and translate the data. Hence, people who have experiences in qualitative data management have done the transcription and translation. Framework and content analysis was used to analyse the data. Framework analysis is gaining popularity in health sciences researches as it is particularly suitable for data’s collected in a cross section to capture various aspects of a phenomenon under investigation [25]. The study employed conventional content analysis, which allows diversity of conceptions and to get richer understanding on access to SBC [24]. In accordance with the procedures in the framework and content analysis, the interview transcripts were first read and re-read several times to get an overall impression of the data. The data were categorized and coded into three major themes and four sub-themes emerged during data analyses.

### Ethical consideration and study permits

The Addis Ababa City Administration Health Bureau ethics committee and the regional ethics Committee in West Norway (REK VEST) have approved the project. Study permits were obtained from Gulele sub-city and Addis Hiwot health center. Before interviews and FGDs, informants were briefed about the objectives of the study and on the procedures on how the interviews would be conducted. They were informed that they had the right to refuse the interview or to stop anytime if they do not feel like it without giving any explanation. Since the women were approached through the urban health extension workers, we made clear to each woman that the interview would have nothing to do with the services they receive from the health extension worker or from health facilities. We used codes to identify informants during analysis and write up. Written consent was obtained from each informant.

## Results

Patterns of service utilization, missed opportunities to postpartum care and accessing and utilizing skilled care at birth were the three major themes emerged when the in-depth interviews and the FGDS were analyzed. The four emerged sub-themes that influence access and utilization of skilled birth care were social influences, perceived risk, physical access to health facility and perceived quality of care and disrespect (Table 1).

**Table 1:** **Themes and sub-themes emerged during the process of analyzing the in-depth interviews and the FGDs data**

**Major themes**	**Sub-themes**
**1. Patterns of service utilization**	
**2. Missed opportunities to postpartum care**	
**3. Accessing and utilizing skilled birth care**	• Social influences
	• Physical access to health facility
	• Risk perception
	• Perceived quality of care and disrespect

### Patterns of service utilization

**Figure 2:**
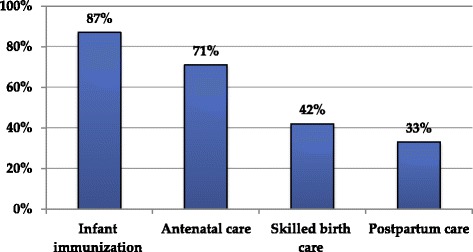
**Proportions of migrant women who utilized antenatal care, skilled birth care and postpartum care and infant immunization in most recent birth in Addis Ababa, Ethiopia.**

Focused ANC is provided in public health facilities in Ethiopia where women should have four ANC visits during pregnancy otherwise necessary with the first visit being in the first trimester. Of the 45 women who participated in the study 32 (71%) women had received ANC in their recent pregnancy (Figure 2). The in-depth interview informants were further asked about the number of ANC visits they had and in which month of their pregnancy they initiated. Eight (44%) women reported that they initiated ANC at first trimester, seven (39%) at second trimester and three (17%) at third trimester while one woman did not remember when she initiated ANC. Two (10.5%) women had one visit, three (15.8%) had two visits, four (21%) had three visits and 10 (52.6%) women had four or more ANC visits.

**Figure 3:**
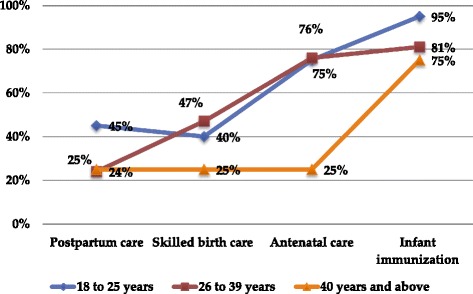
**Patterns of antenatal, skilled birth, postpartum care early infant immunization services utilization in the most recent births across three age groups of migrant women in Addis Ababa, Ethiopia.**

Figure 2 shows patterns of service utilization. Service utilization ranged from 33.3% for PPC to 87.6% for infant immunization. ANC and infant immunization services were fairly equally accessed among informants irrespective of their age and where they gave birth to their last child. There were big access gaps in SBC and PPC services with only 42% and 33% of the informants reported accessing these services respectively. Service utilizations varied across the different age groups of informants where, the age group 18 to 25 years utilized ANC, PPC and infant immunization services more than the other age groups of women (Figure 3).

### Missed opportunities to postpartum care (PPC)

The PPC schedule in the public health centers is at six days and at six weeks afterbirth where both the women and the babies would receive appropriate services. The findings showed missed opportunities to access PPC irrespective of where the women had given birth. Of the 19 (42%) women who received SBC, 8 (42%) women did not receive PPC. Among the 39 (87%) women whose babies had received vaccination within the first six weeks of life, 15 (38%) did not receive PPC. The following quote illustrates the missed opportunities to PPC in a woman who gave birth at home but brought her baby for immunization.

*“I gave birth at home…I took my baby to a health facility for six days immunization and my baby has got vaccination…but they did not check on me. They just advised me to bring my baby for next immunization when he is six weeks old” (Mother of one who gave birth at home)*

### Accessing and utilizing skilled birth care (SBC)

#### Social influences

Social influences were reported as important factor to access and utilize SBC. Although the majority of the informants reported that, the women themselves were the main actor in making decision where to give birth, the influence of significant others such as older generation of women (grandmothers, mothers and mothers in law), husband neighbours/ friends were also mentioned. Women who had strong social support such as close relatives, neighbours and friends around and those who have no education were more vulnerable to social persuasion to deliver home than those women who had little social support and have some education. During the interviews and the FGDs, all generations of women considered home as the right place to give birth although some young and educated informants were contesting this notion. Surrounded by close acquaintances, getting back rub and having someone to hold during labour, having warmth, getting soups and gruel were some of the attributes that many women appreciate during home birth.

*“We Women who have close relatives around us get good care when we give birth at home … we get everything. Even if we get some problems we always have someone to help us. It is always nice to have social support” (Mother of four who gave birth at home)*

On the contrary, women who did not have strong social ties with the community opted for SBC. Most of the informants were migrants from remote rural areas and some of them were actually recently migrated. These recently migrated women who did not integrate well in the community utilized SBC for the most reason that they would not get anyone to attend them at home. A woman with no social support explained her delivery experiences as follow

*“I moved to a new place just before I delivered… I did not know anybody in the area…I suddenly felt labour pain and my husband was not home … since I did not have anybody to could help me at home I decided to go to a health center… I was alone on my way to health center. Unfortunately the labour was very fast and I gave birth outside of my house even before leaving the main compound where I live. The owner of the house helped me to cut the cord, if she was not around my baby would have died” (Mother of two who gave birth at home)*

In spite of other social forces, utilization of SBC was highly influenced by maternal education. According to some of the informants, mothers in laws often try to influence their daughters in law’s decision to receive SBC and could succeed with uneducated daughters in law. Taking an example of a family with two daughters in law, the uneducated daughter-in-law abided by the decision made by her mother-in-law and delivered at home while the educated one made her own decision supported by her husband and gave birth at a health facility despite her mother in law insisting her to have home birth.

Having traditional birth attendants for home delivery was reportedly uncommon in this community. Relatives or neighbours who experienced childbirth themselves were considered as a resource person to assist other women in labour.

#### Risk perceptions

Risk perceptions were reported to play a role in women’s access to SBC. In the case of actual or perceived risks women preferred to access SBC. Intergenerational consensus was reached in this matter that all informants unanimously agreed that the health care providers have the skills and the knowledge to help women with complicated childbirth. Traditional birth attendants and other home attendants were not much appreciated when it comes to the knowledge and the skills they have to handle obstetric and neonatal complications. Many women who had no risks identified during pregnancy and those who had uneventful home births preferred home birth assuming that nothing would happen. The following quote taken from a woman illustrates how having perceived complication alerted her to seek SBC.

*“During my antenatal follow up, the nurse informed me that if I see blood stained vaginal discharge I should seek care at the health facility immediately. When I was in labour the amniotic fluid had different colour than the one I had during my previous births… because of that I went to the health center immediately… from the health center I was referred to hospital” (Mother of four who give birth at hospital)*

Most of the informants said that the health care providers advised pregnant women during their ANC visits to make some saving if in case complications occur then they can use it for hospital bills and for transport bills. Despite receiving advise on birth preparedness, two women who had uneventful home birth in the past said that they did not even save money for transport and hospital fees if birth complications were to happen.

#### Physical access to health facility

In the in-depth interviews and the FGDs physical accessibility of the health facility was reported as a factor for accessing SBC. According to some informants, when the health facility was inaccessible, it was not only delaying the women to reach to health facility but it also made the women reluctant to make early decision to receive SBC. Home birth was much common among women who were living in areas where the walking distance to the nearest health center was more than 15 minutes. These women claimed that it was too far to walk to the health facility for women in labour. The lack of public transport in the area, the cost of transport and the landscape made the health facility more inaccessible to the women. In the following quote a woman who gave birth in a hospital said that she went to hospitals long before labour progressed for fear of not giving birth at home.

*“I am living far from the health center and the hospital. For a woman in labour especially in the evening it is very difficult to reach to health facility … It is not also possible to get ambulance services in our village because of the landscape. During ANC check up the nurse advised me to save money for taxi but not to wait for the ambulance to come as the ambulance would take long time to get to me … I left home walking long before labour progressed not to give birth at home” (Mother of three who gave birth at hospital)*

Some informants reported that it was not in their best interest to give birth at home. But, due to the fast labour progress coupled with the constraints to reach to the health facility they ended up giving birth at home. Three women claimed that they were actually on their way to the health center but labour was too fast that they could not reach, while four women claimed that they were having labour pain in the evening when there was no formal transportation.

*“I had ANC follow ups in the health center during my pregnancy. I did not intend to give birth at home…around midnight I felt rheumatic kind of pain and I didn’t think that it was the labour pain. Since it was also in the evening I did not want to go to health center for something that I thought was not real. Instead I asked my close relative to make the room warm…then labour progressed so fast and I gave birth at home within five hours” (Mother of one who give birth at home)*

#### Perceived quality of care and disrespect

The perceived quality of care at the health facility was reported as the most important factor for accessing and utilizing SBC. In all the FGDs and in the majority of the in-depth interviews disrespectful care was presented as common practice in public health facilities. During the FGD, an elderly woman shared her experience in the following quote

*“Some women in labour pleading us not to take them to health facility for delivery … please don’t take me to health facility. They (the women) would say that the providers in the health facility would shout at them and not treating them with respect. Many women would prefer to die home than to experience bad treatments in the public health facilities” (Elder FGD participant)*

All informants unanimously agreed that women who reported negative experiences in the health facilities either during pregnancy or during previous births often decided to give birth home for fear of humiliation, bad treatment and for not being respected by health care providers. The following quote illustrates why an 18 years old woman with some education who first planned to give birth at the health facility changed her mind in the end.

*“I was thinking to give birth in a health facility… but two weeks before my due date, I was in a health center with a friend who was expecting. The health worker was impolitely asking my friend to do this, to do that while she was in labour pain … imagine. After I saw that, I thought it would also be the same for me and I changed my mind. I said no to my husband when he tried to take me to the health facility during labour” (Mother of one who give birth at home)*

Women who had utilized skilled birth care in previous deliveries reported that the quality of care at the public health centers has shown improvement in their index delivery compared to the past. According to these women, in the past providers were looking down up on labouring women.

*“These days’ things are changing; the health care providers have started properly caring for women in labour. In the past they were yelling at us, they insult labouring women for not being clean. They were saying that why you did not shower, you have bad smell. To avoid this humiliation we were giving birth at home. But now it has been changing” (Mother of four FGD participant)*

According to some of the informants who recently accessed SBC, they appreciated the improvements in the interpersonal communication, providers approach, staying with the women during labour, informing the labouring women what was going on and explaining procedures, being talked politely and with respect.

*“In my previous delivery when I screamed due to the labour pain, the midwives were making fun of me, I guess they were thinking that I was shouting just to disturb them. But with this delivery, they were so different and caring. I experienced shortness of breath when the labour was advancing, for that they put me on oxygen, they cleaned me immediately after birth and they were reassuring me. When I was in labour, they were advising me to sleep on my side…they were telling me about the condition of the foetus. They were even explaining to me what they were going to do before doing any examination” (Mother of two who gave birth at health center)*

Accessing skilled birth care free of charge was reported as another attribute of improved quality of birth care at the primary health centers. Some women reported that, during their past deliveries they were asked to buy gloves and other things for delivery but now everything were available free of charge.

The other aspects of quality according to the women were having friendly services in the labour and delivery wards. According to the interviews with the women, the new strategy, which is making the labour ward friendly to women in labour by having a coffee ceremony in the labour ward, was considered a positive initiative. Two women who received SBC said that creating such atmosphere could help labouring women to relax and to get distracted from the constant labour pain. These women also called for more improvements in the labour and delivery ward such as to keep the rooms warm.

*“Currently you see things in the health centers that would make labouring women comfortable … I could say that it is not less than a home. Unfortunately, I was labouring during the night but if my labour was during the day my families could have prepared coffee in the labour ward. All the necessary things for coffee ceremony were made available in the labour ward. I really liked that …I love coffee. Having this kind of homely atmosphere would help pregnant women to think not only about the labour pain” (A women who gave birth at home)*

According to the informants, despite improvements in the quality of care at the health centers, hospitals continued providing disrespectful and poor quality care. A woman who gave birth at hospital expressed her frustration in the following quote,

*“I still suggest other women to give birth at health facility even though I did not have good experience in the hospitals where I gave birth. The providers at the hospital treated me badly especially the ladies…yet, I feel that my life was saved because I gave birth in the hospitals” (Mother of three who give birth in hospital)*

Three women who referred from health center to hospital for advanced care reported that they were not willing to go to the hospitals. According to these women, the health care providers in the hospitals had negative attitude towards women in labour. Two women reported an inappropriate communication by the health care providers and they were shouted at. While the third woman said that she was rejected and did not get the attention she needed after birth in the following quote,

*“When I first reached to hospital ‘A’ they shocked me by making wrong diagnosis. I was then referred to hospital ‘B’. In hospital ‘B’ I had normal birth around 6:00 in the morning. Then I was taken to another room and my family members were asked to pay the hospital bill. My sister paid the bills… there was no one who came to see me until 7:00 in the evening. I was the only woman who gave birth that day. It was not because they were busy but it I did not know why they ignored me … What I know from my previous delivery was that after the delivery they give medicine for the baby and the mother should send home after four or six hours of delivery. It was strange for me” (A woman who give birth in the hospital)*

Women who reported receiving quality care during pregnancy and in their previous deliveries opted for facility birth. According to accounts of four women, the care they received during ANC follow up or during previous births had attracted them to access SBC in their recent delivery. These informants said that they liked the health care providers and the care at the health centers, as the following quote illustrates

*“In the health center, the midwives were following me closely… they provided me good care. They referred me to hospital when it was beyond their capacity to manage”. (Mother of one who gave birth at hospital)*

Two informants suggested that getting to know the providers who will attend them during labour and delivery beforehand as an important step. They claimed that it would help to build up relationship and trust between women and their providers so that women would not feel that they are in a strange environment during birth.

## Discussion

Migrant women in Addis Ababa have unequal access to SBC and PPC. These inequities are happening in the backdrop of equitable access to and high attendance of ANC, infant immunization and free access to maternal and newborn care in primary care facilities. Physical access to the health facility, social influence, maternal education, risk perception, perceived quality of care and disrespect were reported to be responsible for the disparities to access and utilize SBC. Despite the wide inequities in SBC among migrant women, little attention has given for improving access to SBC the disadvantaged communities in Addis Ababa.

Universal coverage and free access to health care services are some of the strategies recommended to bridge equity gaps in maternal and newborn care services and to minimize delays to accessing lifesaving interventions [19,26]. In recent years Ethiopia has succeeded in increasing health services coverage to its people. This success story follows the rolling out of the rural health extension strategy that enables to reach out the hardest-to-reach remote villages and communities including migrant population [13]. Similarly, Addis Ababa, having 100% coverage of maternal and newborn care services uses the urban health extension programme as a platform to respond to the health need of households. Health care financing reforms are the other initiative that the government of Ethiopia is implementing for ensuring equitable access to maternal and newborn care services [16-18]. The reforms assert fee exemption to maternal and newborn care services at primary care facilities irrespective of the women’s willingness to pay and migration status. The informants in the present study acknowledged the free maternal and newborn care services in the public primary health centers, but because of the economic disadvantages some women would have hesitated to access needed care at the hospitals where they have to pay. In a qualitative account of internal migrants in Shanghai, China, it has shown that women prefer home birth for the costs of hospital bills [19].

The poor access to SBC in the backdrop of universal and free access to health care services would suggest the need for tailored interventions to address barriers and special need of these disadvantaged and marginalized migrant women in the community. Without addressing structural drivers responsible for the unequal access and unequal opportunities, achieving equity in SBC would be unrealistic [26]. The Addis Ababa City Administration Health bureau, Gulele Sub-city health bureau and concerned stakeholders should have a thorough understanding of the underlying determinants of the disparities in intrapartum care utilization in these communities. Access to SBC was reportedly hindered by distance to the health facility in the present study. This problem was aggravated by the fast labour progress and having labour being established in the evening. Studies from resource poor setting, including Ethiopia have shown that access to maternity care is affected by distance to health facilities and place of residence, where improving transport with voucher system has increased service uptake [27-31]. Concerned bodies should consider having dedicated ambulance services for transporting labouring women to and from the health facilities 24 hours per day and seven days per week until sustainable solutions are instituted.

According to the present study, access to ANC and infant immunization was more equitable compared to access to SBC and PPC. Several quantitative studies reported similar findings that ANC and infant immunization are the most equitable services while SBC is the most inequitable intervention in Ethiopia [14,30]. In this study, it was described that having social support was a factor that influence women decision to seek SBC while women who have no education appeared more vulnerable than women with some education. Consistent with our findings, two studies from Ethiopia and a review show that social persuasion in making decision to seek SBC is less for empowered and educated women [9-11]. According to the Ethiopian DHS 2011 report, education was an important determinant to access and utilize perinatal care services [13]. Studies have shown that pregnant migrant women often have low education and low economy and this might increase their social vulnerability [19,32]. Improving the status of women in the community through education, involving them in self-help groups and income generation activities and encouraging their participation in community conversation would have an immense return for improving access to SBC and for narrowing the equity gaps.

In the present study, actual or perceived risks were compelling reasons for seeking SBC. Among the informants, there was intergenerational census that health care workers are experts who can handle childbirth complications. Consistent with our findings, several studies from Ethiopia [9,10,33] and a literature review [34] show that women who reported to have complications in previous births often utilize SBC, where similar experiences are also reported among internal migrant women in China [19].

Consistent with findings from other studies [11,31,35-37], the perceived quality of maternal and newborn care services plays a significant role in women’s decision to access SBC. A study from Tanzania shows that high quality ANC has increased uptake of SBC [37]. In the present study, respectful care was the core element when informants were assessing the service quality. According to the informants account, disrespectful care is commonplace and many women either experienced it or have witnessed such behaviours. Health providers’ attitude, reception and care during labour and delivery were reportedly unpleasant that discouraged many pregnant women from accessing SBC although improvements have been reported in recent years. Studies from different regions in Ethiopia have documented experience of disrespectful care when women seek SBC and also highlighted how such experiences are pushing the women away from seeking SBC [9,10]. Recently, several studies from different parts of Africa have uncovered the widespread practices of disrespectful and abusive care and its potential consequence in reducing access and utilization of SBC [7,9-11,38].

The current initiative emphasizing on demand creation for skilled birth care using urban extension workers would backfire if the service qualities at the health facilities remain sub-optimal and disrespectful. Although, quality issues have gained momentum in recent years, there appears to be a lack of recognition that improving service quality could also bridge equity gaps in SBC. To ensure improvement in the overall quality of care considerations should be made in creating awareness on women’s reproductive health right and the right to receive respectful care, revisiting professional code of conduct, establishing a system of accountability, improving providers’ competence and performances, addressing logistic challenges, devising and implementing appropriate strategies to identify and address disrespect in maternal and newborn heath care settings.

ANC is an entry point for women to access conception, intrapartum and postpartum interventions, where a recent review highlighted its significance for improving maternal and neonatal survival [39]. Yet, an increasing body of knowledge has revealed gaps on the implementations of focused ANC, although coverage’s are high the qualities and contents of the services are sub-standard [40,41]. A mixed methods study in Ethiopia reports that 40% of pregnant women are not satisfied with the overall quality of the focused ANC they received [42]. In a study in Zimbabwe that analysed Demographic and Health Survey data, only 29% pregnant women received quality ANC with most of the contents well addressed [40]. This appears to be the case in the present study; despite almost all the in-depth interview informants receiving ANC, few women were aware of the need to have PPC. This seems to have contributed to the missed opportunities to PPC. Although most of the informants in this study had visited health facility at six days and six weeks for infant vaccination only few women actually received PPC.

The missed opportunities to PPC could also be linked to poor integration of the maternal, newborn and childcare services within the health facilities. Proper integration of these services is essential to make sure that all women who bring their babies for immunization also receive PPC. Moreover, extending the role of the urban health extension workers to deliver home based services for women who fail to come for PPC could be an area to be looked upon. The urban health extension workers are nurses by profession who additionally received three months training on health extension programme. These health cadres fall in the category of skilled attendants, according to the standards set by the WHO and the FMOH, yet currently their role is limited to creating demand for pre-conception, pregnancy, SBC and PPC services.

This is one of the first study exploring inequities in SBC among internal migrant women in Addis Ababa. The findings of the study would be valuable for the city administration, health bureau and concerned stakeholders to see from the equity dimensions for the low utilization of SBC among marginalized migrant women. Several studies in Ethiopia have reported inequities in access to maternal health care services. The present study would add some knowledge about the disparities to access SBC by exploring the underlying factors for the inequities using informants’ qualitative accounts. The study captured perspectives on facilitators and barriers to access SBC among women who gave birth at health facilities and those who gave birth at home. To improve credibility of the study, member checking and triangulation of data collection methods were used. The findings of the study could be transferable to other urban settings within or outside of the country that are experiencing fast economic growth leading influx of large rural population seeking for better livelihood opportunities. The present study is limited to a single migrant population in the city where there could be social and cultural differences among other migrant populations across the city, which potentially affect care-seeking behaviours. Since structural and health system factors were reported as the major barriers to access SBC by the informants, the situation among other migrant population would not be much different.

## Conclusion

Migrant women constitute disadvantaged communities in Addis Ababa and have unequal access to SBC. This happens in the backdrop of equitable access to and high attendance of ANC, universal health service coverage and free access to maternal and newborn care in primary health facilities. If the city is to achieve improvement in maternal and newborn care, addressing the underlying determinants for the inequity in SBC and improving the quality of maternal and newborn care for migrant population should be prioritized. The interplay of structural, health system and social factors for the observed inequities are calling for multi-sector responses beyond the health sector. On top of the structural barriers, disrespectful care was presented as a common encounter in the health facility when women were to access SBC and is serving as a strong disincentive to SBC. In line with the recent WHO calls for action, Ethiopia should work on having strategies to document, prevent and address the rampant practice of disrespectful care in maternity care setting if the country is to achieve equitable access to SBC.

Concerned stakeholders would consider extending the role of urban health extension workers to deliver PPC at home settings and minimizing missed opportunities to PPC at the health care facilities. Improving women’s status in the community could be one way to address some of the structural barriers responsible for the unequal access to maternal and newborn care services among these populations. The health sector should keep pace with the rapid economic development in the country accruing social transformation through regular revisions and adaptation of various strategic approaches for improving maternal and neonatal health outcomes.

## Additional file

Additional file 1:
**In-depth interview guide.**

## Abbreviations

ANC: Antenatal care
DHS: Demographic and Health Survey
FGD: Focus group discussion
FMOH: Federal Ministry of Health
PPC: Post-partum care
SBC: Skilled birth care
WHO: World Health Organization
