# Supplementary material for: Exploring inequities in skilled care at birth among migrant population in a metropolitan city Addis Ababa, Ethiopia; a qualitative study
Source: Int J Equity Health. 2014 Nov 25;13:110. doi: 10.1186/s12939-014-0110-6 (PMC4246478; doi:10.1186/s12939-014-0110-6)
Supplement: Additional file 1: — In-depth interview guide. [file 12939_2014_110_MOESM1_ESM.pdf]

In-depth interview guide

I. Social and demographic characteristics

- o Age __________
- o Religion 1. Orthodox Christian 2. Muslim, 3. Protestant Christian 4. others ____________
- o Marital status 1. Married, 2. Never married, 3. Divorced, 4. Widow
- o Family structure 1. Nuclear family 2. Extended family
- o Are you living with your mother in law? 1. Yes 2. No
- o Education _____
- o Occupation 1. Weaving 2. Selling fuel wood 3. Housewife 4. Others ________

II: Obstetric history and service utilization

- o Gravidity (number of pregnancy) ______________
- o Did you ever have an abortion 1. Yes 2. No if yes, how many times _________
- o Parity (number of delivery)_____________
- o Did you have antenatal care visits during pregnancy? 1. Yes 2. No
- o In which month of your pregnancy you started attending antenatal care? ______
- o How many times did you visit antenatal clinic before delivery? _________
- o Did you have any problems or complication during pregnancy? 1. Yes 2. No
- o If yes, please specify? ______________________________
- o Date of delivery ______________
- o Place of delivery 1.home, 2.health centre, 3.hospital
- o Have you received postpartum care in the first six weeks after birth? 1. Yes 2. No
- o Has your baby received vaccination in the first six weeks after birth? 1. Yes 2. No

III. Childbirth experiences of woman who gave birth at home

- Why you chose to give birth at home? (Probes: no pregnancy complication, no complication in past deliveries, not informed by health care workers, living far from health facility, labour occur at night, no one to take me to health facility,

negative experiences with the health providers during pregnancy and during past delivery, prefer home care, family support during home birth)

- Who made the decision? (Probes: yourself, husband, relatives, friends, neighbours)
- Who were by your side during labour?
- Who attended the delivery?
- What cultural practices were made to celebrate the birthing process? (Probes: for the mother, for the baby, for the placenta)
- What other cultural practices could have happened if you had all the necessary resources?
- What was the role of your husband/friends/relatives/neighbours in the birthing process?
- Did you have any complications during birth? 1. Yes 2. No
- If yes, tell me what kind of complications you had and how it was manage?
- What kind of immediate care was given to you and to your baby after birth?
- Comparing the delivery care given to women where you came from, how do you see the birth care that you received?
- If you were to get pregnant again where would you like to give birth? (Probes: would you make special preparation?
- Where would you advice your friends to give birth?

IV. Childbirth experiences of woman who gave birth at health facility

- Why you chose to give birth at health facility? (Probes: pregnancy complication, complication in past deliveries, informed by health care workers, living near health facility, positive experiences with health providers during pregnancy and during past delivery)
- Who made the decision? (Probes: yourself, husband, relatives, mother in laws, her mother, friends, neighbours)
- What kind of support you received from significant others with respect to seeking birth care in health facility?
- How did you reach to the health facility? (Probes: walking, carried by people, taxi, ambulance)

- Could you remember how long time you spent in the health facility before you received care (Probe: received prompt attention)
- How was the communication with the midwives? (Probes: informed about procedures and progress of labour, expressed your concerns, did they listen to you, an opportunity for you to pose questions)
- What do you think about the care that you received?
- How did the health workers treat you? (Probes: treated with respect, talked to with kindness, protected privacy, instructed in an understandable manner).
- Was your family allowed to stay with you during the first phase of labour?
- Would you tell me the kind of care you have received during birth? (Probes: for you, for the baby, the placenta)
- Did you have any complication during labour and delivery? If yes, could you please describe what happened and how you were treated? Where you happy with the care?
- What would you have liked to have during labour and delivery if you were allowed to do so in the health facility?
- What was the role of your husband/friends/relatives/neighbours in the birthing process?
- How long did you stay in the health centre/hospital after birth?
- Who accompanied you when you went back home?
- During your stay in the health facility:-
    - What did you consider good care?
    - What did you consider not so good care?
    - How did you see the attitudes of the midwives?
    - How would you like to be treated/expect to be treated during labour and delivery?
    - What would you suggest that the health workers could do differently in order to make a woman in labour feel safe and respected?
- If you were to get birth again where would you like to give birth? Why?
- Where would you advice your friends to give birth? Why?
